# Impact of Human Management on the Genetic Variation of Wild Pepper, *Capsicum annuum* var. *glabriusculum*

**DOI:** 10.1371/journal.pone.0028715

**Published:** 2011-12-06

**Authors:** Pablo González-Jara, Alejandra Moreno-Letelier, Aurora Fraile, Daniel Piñero, Fernando García-Arenal

**Affiliations:** 1 Centro de Biotecnología y Genómica de Plantas, E. T. S. I. Agrónomos, Campus de Montegancedo, Universidad Politécnica de Madrid, Pozuelo de Alarcón (Madrid), Spain; 2 Departamento de Ecología Evolutiva, Instituto de Ecología, Universidad Nacional Autónoma de México, México City, México; University of Lausanne, Switzerland

## Abstract

Management of wild peppers in Mexico has occurred for a long time without clear phenotypic signs of domestication. However, pre-domestication management could have implications for the population's genetic richness. To test this hypothesis we analysed 27 wild (W), let standing (LS) and cultivated (C) populations, plus 7 samples from local markets (LM), with nine polymorphic microsatellite markers. Two hundred and fifty two alleles were identified, averaging 28 per locus. Allele number was higher in W, and 15 and 40% less in LS and C populations, respectively. Genetic variation had a significant population structure. In W populations, structure was associated with ecological and geographic areas according to isolation by distance. When LM and C populations where included in the analysis, differentiation was no longer apparent. Most LM were related to distant populations from Sierra Madre Oriental, which represents their probable origin. Historical demography shows a recent decline in all W populations. Thus, pre-domestication human management is associated with a significant reduction of genetic diversity and with a loss of differentiation suggesting movement among regions by man. Measures to conserve wild and managed populations should be implemented to maintain the source and the architecture of genetic variation in this important crop relative.

## Introduction

Biodiversity provides numerous services, one among them being food production [1], [2]. Plant species that are exploited by humans may be under specific risks of diversity loss, associated with changes in their ecology along a continuum, leading from unmanaged exploitation of wild populations to silvicultural management, cultivation and domestication. Genetic drift associated with population bottlenecks during domestication and selection of favourable traits results in a loss of genetic variation [3, 4, and 5]. Domestication of major crops occurred thousands of years before present (BP), and the effects of domestication on genetic variation can only be analysed by historical reconstructions based on data from present wild and domesticated populations. However, domestication of native plants is an ongoing process, particularly active in Mesoamerica [6], [7], [8], one major centre of plant domestication [9], [10]. Current human management of plant populations and incipient domestication provide an opportunity to study the effects of these processes on plant genetic variation, which is highly relevant for conservation biology and for understanding past and ongoing domestication processes. This is the goal of this work, which focuses on a crop relative, the wild pepper *Capsicum annuum* var. *glabriusculum* (Dunal) Heiser and Pickersgill syn. *C. annuum* L. var. *aviculare* (Dierbach) [11], also known as “chiltepin”.

*C. annuum* var. *glabriusculum* is considered the ancestor of the cultivated chili and bell pepper, *C. annuum* var. *annuum* [12], [13], which is the most economically important domesticate of five *Capsicum* species. *C. annuum* var. *glabriusculum* is a perennial bush distributed from Colombia to the South Western United States [14]. In Mexico, it can be found from the Yucatan peninsula and the Gulf of Mexico, where it grows in deep soils with dense evergreen vegetation, to xeric regions in the Sonoran desert or the central plateau, where it is commonly associated with nurse trees [15]. Fruits of chiltepin are consumed by birds, which act as dispersal agents [15]. Chiltepin has high phenotypic plasticity, shown by the variation of traits such as leaf morphology, fruit shape, pattern of seed germination or resistance to pathogens like *Pepper huasteco yellow vein virus* [16]. Archaeological remains document the human use of wild pepper as part of the diet since 8000 BP [17]. Harvesting the fruits of chiltepin is still a common practice in central and northern Mexico and the total harvest has been estimated to be 50 metric tonnes per year [18]. In some areas such as the Sonoran desert, overexploitation might have proceeded for several decades, and has been identified, together with habitat loss, as a cause of decline and even extinction of local populations [19]. In addition to fruit gathering from wild populations, human exploitation of chiltepin involves let standing of chiltepin plants in pasture lands (potreros) and living fences, as well as cultivation in home gardens. In very recent years the cultivation of chiltepin has progressed to monocultures in small traditional fields, possibly as a result of growing demand and recession of their wild populations [20]. In cultivation, chiltepin is managed as an annual crop. Cultivated chiltepin does not show obvious phenotypic differences in comparison with wild populations. It has the characteristic small, red, erect and deciduous pungent fruits of wild chiltepin [11], and does not present the major traits of pepper domestication syndrome, which are large, pendulous, non-deciduous fruits of different colours and pungency, flower morphology favouring selfing, and synchronised high germination rates [21]. Thus, chiltepin provides a system to analyse the effects on the genetic diversity of a native plant of very early stages of human management, potentially leading to domestication. Genetic analyses of one *C. annuum* var. *glabriusculum* population from Arizona (USA) and of *ex situ* accessions from Sonora, Chihuahua (Mexico) and Guatemala found genetic variation that was absent from other *C. annuum* accessions [18]. Other genetic analyses of *C. annuum* have found a slightly higher variation in wild chiltepin population than in landraces or varieties of domestic *C. Annuum* var. *annuum* [22]–[24]. Also, a high population structure, probably due to geographic causes, was reported for wild chiltepin populations from Sonora and Sinaloa, in NW Mexico [24].

The analysis of the effect of human management on the genetic variation and structure of chiltepin based on the study of both wild and managed populations at a large geographic scale has not been undertaken. This is the subject of this work. We sampled neighbouring wild, let standing and cultivated chiltepin populations (*sensu* Casas *et al*. [25]) from several contrasting geographic regions in Mexico. We used microsatellite markers to examine whether human management results in changes in the genetic diversity and structure of chiltepin populations, and to detect possible demographic bottlenecks in the recent past. Our results show a significant decrease in chiltepin population size in the last few thousand years, probably due to habitat fragmentation. We also present evidence that cultivation, although recent, has resulted in a significant decrease in genetic variation and in the breakdown of the genetic structure of wild populations.

## Materials and Methods

### Ethics statement

This work did not involve endangered or protected species.

### Plant Collections

**Figure 1:**
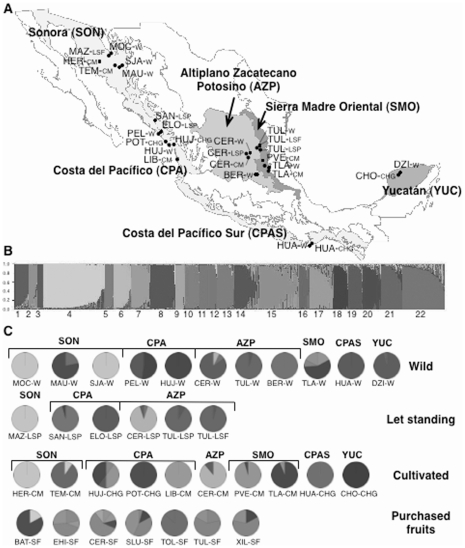
Geographic location, population structure and genetic composition of *C. annuum* var. *glabriusculum* populations. (A) Location of populations from wild, let standing and cultivated habitats within six biogeographical provinces in Mexico. (B) Six hundred and sixty one genotyped individuals from all populations clustered into 22 groups. Each individual is represented by a vertical bar which is divided into 22 coloured fractions representing the estimated portion of its genome that assigns the individual to each of 22 clusters. Black lines separate different clusters. (C) Each wild, let standing, cultivated and local market population is represented as a pie chart showing the proportion of individuals assigned to each of 22 clusters and the biogeographical province of origin, following the same colour coding.

Chiltepin plants were sampled during the summers of 2007–2009 at different sites over the species distribution in Mexico (Fig. 1A). A total of 27 populations were sampled in different habitats representing three levels of human management: i) eleven wild (W) populations in which fruit gathering by local people may occur; ii) six let standing (LS) populations (*sensu* Casas *et al*. [25]) in anthropic habitats, either pastures (LSP) or live fences (LSF), in which chiltepin plants are tolerated or favoured, and iii) ten cultivated (C) populations in either home gardens (CHG) or small monocultures (CMC). Population sites were assigned to 6 biogeographical provinces: Yucatan (YUC), Eastern side of the Sierra Madre Oriental (SMO), Altiplano Zacatecano-Potosino (AZP), Costa del Pacífico (CPA), Costa del Pacífico Sur (CPS) and Sonora (SON) [26]. Relevant information on these populations appears in Table 1. At each population one plant out of every *x* plants was sampled along fixed itineraries, itinerary length and *x* (0<*x*≤4) depending on the population size. Between 1 and 3 young branches with fresh leaves were collected per plant.

**Table 1:** Summary of collection data for 34 Mexican *C. annuum* var. *glabriusculum* populations.

Location^1^	Code^2^	Region^3^	Habitat	Latitude	Longitude	Elevation^4^
**Field populations**						
Dzibilchaltun (YUC)	DZI-W	YUC	wild	21.092	−89,595	9
Cholul (YUC)	CHO-CHG	YUC	home garden	21.053	−89,558	9
Huatulco (OAX)	HUA-W	CPS	wild	15.795	−96,053	15
Huatulco (OAX)	HUA-CHG	CPS	home garden	15.800	−96,055	19
Tlacuapa (SLP)	TLA-W	SMO	wild	21.418	−98,945	510
Tlacuapa (SLP)	TLA-CMC	SMO	monoculture	21.417	−98,947	550
PuertoVerde (SLP)	PVE-CMC	SMO	monoculture	21.912	−99,423	869
Tula (TAM)	TUL-W	AZP	wild	23.001	−99,659	1244
Tula (TAM)	TUL-LSF	AZP	live fence	22.979	−99,628	1262
Tula (TAM)	TUL-LSP	AZP	pasture	22.994	−99,648	1270
Bernal (QRO)	BER-W	AZP	wild	20.910	−99,826	1793
Cerritos (SLP)	CER-W	AZP	wild	22.451	−100,239	1170
Cerritos (SLP)	CER-LSP	AZP	pasture	22.449	−100,244	1184
Cerritos (SLP)	CER-CMC	AZP	monoculture	22.448	−100,244	1144
La Libertad (NAY)	LIB-CMC	CPA	monoculture	21.593	−105,173	149
El Potrero (SIN)	POT-CHG	CPA	home garden	23.391	−106,448	36
El Huajote (SIN)	HUJ-W	CPA	wild	23.106	−106,116	48
El Huajote (SIN)	HUJ-CHG	CPA	home garden	23.127	−106,057	51
Puente Elota (SIN)	PEL-W	CPA	wild	23.954	−106,726	92
Elota (SIN)	ELO-LSP	CPA	pasture	24.016	−106,702	139
Sanalona (SIN)	SAN-LSP	CPA	pasture	24.791	−107,136	122
San Javier (SON)	SJA-W	SON	wild	28.600	−109,716	796
Moctezuma (SON)	MOC-W	SON	wild	29.571	−110,002	1129
Mazocaui (SON)	MAZ-LSF	SON	live fence	29.528	−110,126	491
Los Mautos (SON)	MAU-W	SON	wild	28.635	−110,188	437
Temporal (SON)	TEM-CMC	SON	monoculture	28.715	−110,351	352
Hermosillo (SON)	HER-CMC	SON	monoculture	29.013	−111,134	211
**Local markets**						
Xilitla (SLP)	XIL-LM	SMO				
Tula (TAM)	TUL-LM	AZP				
Tolimán (QRO)	TOL-LM	AZP				
San Luis Potosí (SLP)	SLU-LM	AZP				
Cerritos (SLP)	CER-LM	AZP				
Escuinapa Hidalgo (SIN)	EHI-LM	CPA				
Batopilas (CHI)	BAT-LM	SON				

1 State is indicated in parenthesis^:^ CHI =  Chihuahua; NAY = Nayarit; OAX =  Oaxaca; QRO =  Querétaro; SIN  =  Sinaloa, SLP =  San Luis Potosí, SON =  Sonora, TAM =  Tamaulipas, YUC =  Yucatan.

2 Populations are named with the three first letters of the name of the nearest village, plus a code indicating the habitat: W =  wild, LSP =  Let standing, pasture; LSF =  Let standing, living fence; CHG =  Cultivated, home garden; CMC =  Cultivated, monoculture; LM =  Seeds from fruits purchased at local markets.

3 YUC: Yucatan; CPS: Costa del Pacífico Sur; SMO: Sierra Madre Oriental; AZP: Altiplano Zacatecano-Potosino; CPA: Costa del Pacífico; SON: Sonora.

4 meters above sea level.

A second set of samples came from seeds contained in ripe chiltepin fruits purchased at local markets in the same regions where field surveys were conducted (Table 1). In all cases the people selling the fruits claimed that they had been collected from wild local chiltepin populations. Seeds were germinated and grown in a greenhouse under 16 h light /8 h dark cycle at 25°C prior to tissue collection. For analyses, plants derived from a single batch of purchased fruits were treated as a population, identified as LM.

### DNA extraction and genotyping

Total nucleic acids were extracted by grinding 200 mg of fresh leaf tissues in three volumes of 200 mM Tris-HCl pH 9, 25 mM EDTA, 1% SDS, 400 mM LiCl, followed by phenol-chloroform extraction. A set of 10 nuclear microsatellites markers (CAMS-020, CAMS-336, CAMS-351, CAMS-405, CAMS-424, CAMS-460, CAMS-806, CAMS-811, CAMS-844 and CAMS-885) were selected on the basis of their genetic variation in *C. annuum* cultivars: loci with high and low number of alleles were included, and the ten loci belonged to different linkage groups [27]. Microsatellite loci were amplified by PCR using a forward primer labelled with one of the dyes 6-FAM, NET, PET or VIC (Perking Elmer Applied Biosystems) following the touchdown conditions described previously [27]. PCR products were run in an ABI PRISM 3700 Genetic Analyzer using Gene Scan-500-LIZ as a marker size (Applied Biosystems), their size was determined by the Peak Scanner v1.0 Software (Applied Biosystems), and alleles were recorded as the closest size due to the presumed motif repeats. CAMS-811 failed to amplify in 29% of individuals, all of them belonging to populations located in CPA or in SON, suggesting that this locus might contain additional polymorphism within a primer sequence or null alleles. Among the 9 remaining microsatellites average frequency of missing data was 0.4%. Therefore, comparisons that involved all plant populations were based on the 9 loci that amplified in all populations and the DNA profiles that include missing data do not contain more than one locus per individual.

### Population genetic analyses

Genetic variation was measured as the mean number of alleles sampled (*N*_a_), unbiased expected heterozygosity (*H*_S_) [28], observed proportion of heterozygotes (*H*_o_) and allelic richness (*R*_S_) [29]. Inbreeding coefficient (*F*_IS_) was also estimated for each population. These parameters were estimated using FSTATv.2.9.3 [30]. The number of multilocus genotypes (MG) and private alleles (PA) were detected by means of GenAlEx [31]. The amount of genetic differentiation between pairs of populations was estimated by *F*_ST_ [32] not assuming Hardy-Weinberg equilibrium within populations as implemented in FSTAT. To take into account the effect of high diversity on differentiation measures, *D_est_* was estimated using SMOGD v1.2.5 [33], [34]. An exact test in FSTAT was performed to test for linkage disequilibrium between pairs of loci. The significance of these tests was assessed with 1000 random permutations. Stepwise Analysis of Molecular Variance (StAMOVA) was used to take into consideration the effects of the covariates latitude and longitude when decomposing the genetic variance for biogeographical province, level of human management and population [35], [36]. Genetic relationships among populations were determined in two different ways. First, Population Graphs were used [37] to represent relations among populations. Population Graphs use distances between individuals or populations to construct a network of nodes, edges linking nodes being proportional to population covariances. The resulting network leaves populations with an independent structure unconnected. This method is nonhierarchical and allows for reticulate relationships. In the present system this is a reasonable approach since we are particularly interested in identifying populations that serve as bridges to gene flow. In a second approach, the model-based genetic clustering algorithm implemented by Structure [38], [39] was used to infer the number of clusters (*K*) in the whole data set and to confirm the putative geographic origin of the alleles of sampled individuals. This is especially relevant in the case of samples from anthropic habitats or those from fruits acquired in local markets, in which translocations may occur. To this end, microsatellite genotypes were analysed using the admixture model with correlated allele frequencies and without prior geographic information. The algorithm was run with 40000 Markov Chain Monte Carlo (MCMC) iterations of burn-in length and 40000 after-burning iterations for parameter estimation. The number of ancestral populations (*K*) was determined by doing 10 test runs with *K* values ranging from 10 to 36. The final *K* value was chosen based on the likelihood value that was significantly higher than the *K*-1 values using a Wilcoxon test. With the chosen *K* value, 20 more runs were performed with 60000 MCMC length and 60000 after-burning iterations. Individuals were organized in clusters as indicated by the run of the largest likelihood from the most probable value of *K.* The analysis of the most probable number of ancestral clusters within wild populations was performed using the same method but for *K* values from 2 to15, again with 10 runs of the algorithm.

Mantel correlation tests to assess the relationship between geographic and genetic distance matrices were performed using the isolation by distance (IBD) web service (http://ibd.sdsu.edu/~ibdws/) [40]. A matrix of geographical distances between pairs of populations was obtained by using GenAlEx6 [31]. Geographical distance, Neís *D_A_* genetic distance and *D*_est_ distance values between pairs of populations were log transformed, and to assess the correlations 1000 permutations tests were carried out. For the analyses, only data from wild populations were used as the input on the following sets: 1) all populations, 2) populations from the Western provinces CPS, CPA and SON, and 3) populations from the Eastern provinces YUC, SMO and AZP. In addition, for each W population, a spatial structure analysis was done using Structure. SJA-W was excluded from this analysis due to lack of data on the exact spatial position of sampled individuals in the field, and CER-W and HUJ-W because data were available for less than 20 individuals. The number of subgroups within each wild population was inferred in the same way as described above for *K* values ranging from 2 to 7.

To explore historic demographic changes and possible bottlenecks, the coalescence-based MCMC method implemented by MSVAR 1.3 [41]–[43] was used. In this, a stepwise mutation model is assumed for microsatellite loci, and the posterior probability distribution of demographic parameters is estimated using MCMC simulations based on the observed distribution of microsatellite alleles [41]. The important output parameters are (i) *N*_0_, the current effective number of individuals and *N*_1_, the effective number of individuals at the time where the expansion/decline began. In a declining population, *N*_0_/*N*_1_ is smaller than 1 (ii) *t_a_*, the number of years since the beginning of the expansion/decline and, (iii) *µ*, the mutation rate. This approach combines information from all loci for parameter estimation. The analyses were performed using the exponential growth model which is more suitable for modeling changes in population size on a shorter time scale [41], and was run on each cluster of W populations as resulted from Structure with three independent replications using different starting values. Each run for each lineage consisted of 2×10^8^ steps and was sampled every 10000 steps. Posterior density from individual runs was examined to check for overall consistency in shape, using Tracer 1.5 [44]. This software was used to estimate modes and credibility intervals. Plots of the parameters of interest were always similar across the replicates and unimodal, providing a strong indication that the Markov Chain had converged [41]. For each population or group of populations, priors for the first run were: *N*_0_ = 1×10 ^3^, *N*_1_ = 1×10 ^5^, mutation rate (*µ*) = 1×10^−4^ and *t_a_* = 1×10^3^; for the second run were: *N*_0_ =  1×10^3^, *N*_1_ =  1×10^4^, *µ* = 1×10^−4^, *t_a_*  =  1×10 ^3^; for the third run were: *N*_0_ = 1×10^3^, *N*_1_ =  1×10^5^, *µ* =  1×10^−4^, *t_a_*  =  1×10^4^. Results of these runs are presented combined as obtained with LogCombiner 1.4.8 [44]. A 4-year generation time was assumed based on unpublished demographic data obtained by us between 2007 and 2010.

### Statistical analyses

General linear mixed models were used to compare the within population genetic variation according to the level of human management. Plants derived from the seeds in fruits from local markets were not taken into account for these analyses. Estimates of *H*_S_ and *R*_s_ include a correction for uneven sample sizes, but the value of *N*_a_ is expected to increase with the number of individuals analysed. Thus, a correction for sample size was included in the models for comparison of *N*_a_. To explore inbreeding levels with respect to levels of human management, comparisons of *F*_IS_ were carried out. To test whether the different levels of human management affect the genetic variation, the fixed effect of level of human management (W, LS or C) on the mean within population variation was analysed considering locus, population and biogeographical province as random effects. *N*_a_ was transformed by the square root to meet the criteria of normality and homocedasticity. The remaining dependent variables were used without transformation. Analyses were performed with the JMP7 software (SAS Institute, Cary, NC, USA).

## Results

### Genetic variation in chiltepin

A total of 661 individuals were genotyped, 228 from wild habitats, 284 from anthropic habitats (LS and C), and 149 from seeds of fruits from seven LM. Marker CAMS-811 did not amplify in most individuals from SON and CPA populations. For the other populations, 33 alleles were found at this locus. The other nine microsatellite markers were amplified in all populations; the number of alleles per locus ranged from 7 (CAMS-020) to 102 (CAMS-885), averaging 28.6±27.2 alleles per locus. A total of 589 different genotypes were detected for 9 loci. Identical multilocus genotypes were frequent in some populations from anthropic habitats like LIB-CMC and POT-CHG, and extreme in HUA-CHG where all alleles were fixed for ten genotyped individuals. Mean number of alleles per population ranged from 1.00 to 11.11. Private alleles were observed in 17 populations, including 3 C populations, and plants from seeds from three LM. The number of private alleles varied from one allele in a single locus in SJA-W, MAU-W and TEM-CMC (with frequencies between 0.022 and 0.068) to 16 alleles distributed in six loci in DZI-W (frequencies between 0.022 and 0.196). The highest genetic variation, estimated by the sample size-corrected statistics *R*_S_ and *N*_a_ was found in populations from YUC, and the lowest in populations from SON (*F*_(5,18)_> 4.10, *P*< 0.012).

The same trends were observed with *H*_o_ and *H*_s_ estimates. Conversely, *F*_IS_ was highest in SON and lowest in YUC (*F*_(5,17)_  =  3.63, *P*  =  0.02). The generally high *F*_IS_ values most probably indicate high rates of selfing.

### Genetic structure among chiltepin populations

**Figure 2:**
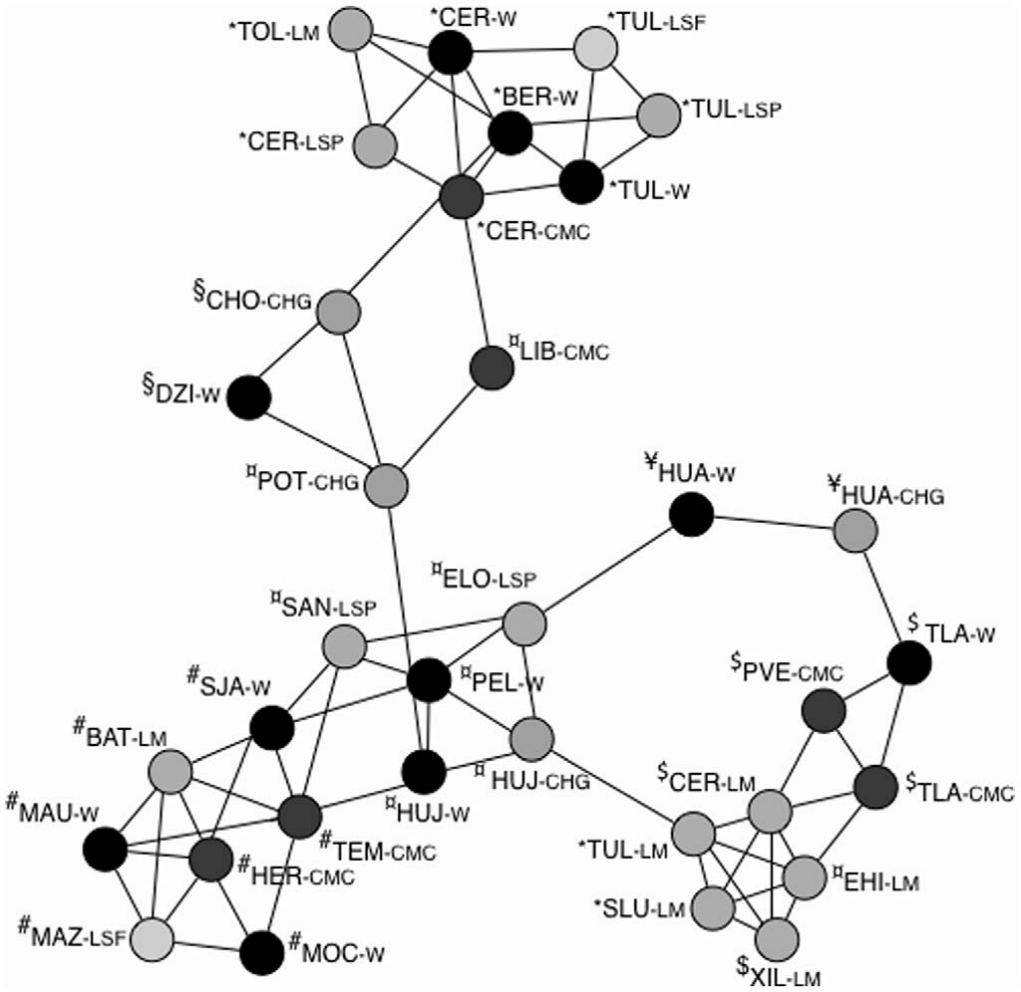
Genetic relations among chiltepin wild, let standing, cultivated and local market populations represented with a Population Graph. Edge length represents the among population genetic variation. No connectivity means no covariation and migration. The origin in six biogeographic provinces in Mexico of the wild populations within each cluster is shown with the following symbols. §, YUC  =  Yucatan, $, SMO  =  Sierra Madre Oriental, *, AZP  =  Altiplano Zacatecano Potosino, ¥, CPS  =  Costa del Pacífico Sur, ^¤^, CPA  =  Costa del Pacifico, #, SON  =  Sonora, £ SIN  =  Sinaloa. Colours correspond to different habitats; Black, wild, Red, cultivated, Yellow, live fence, Fucsia, pasture, Green, home garden, Blue, local markets.

A graph depicting the relations among populations using variance-covariance relations among the 27 field populations and seven LM populations showed significant geographical clustering (Fig. 2). When populations were grouped according to level of human management, no clear subgroups could be recovered except for five of the LM collections (XIL-LM, TUL-LM, SLU-LM, CER-LM and EHI-LM), whose subgraph was significantly disconnected from the rest (P < 0.0033) [37]. The Population Graph obtained with all populations showed high connectivity (Fig. 2). When W populations and LS+C populations were analysed separately, disconnected graphs were obtained (Fig.S1, A and B). This result suggests that gene flow among unconnected W populations only occurs through LS and/or C ones. Populations from each of the four biogeographical provinces of YUC (P < 0.0444), CPS (P < 0.0183), SMO (P < 0.0089) and AZP (P < 4.28×10^−7^) formed significant subgraphs (Fig.2), while populations from CPA and SON were together in a significant subgraph (P < 7.01×10^−9^). Populations from YUC connect AZP populations with the rest. Interestingly, most LM populations grouped with populations from SMO, and not with populations from regions where fruits were purchased, exceptions being TOL-LM and BAT-LM (Fig. 2). These results suggest geographical translocation of chiltepin fruits for market selling and cultivation, which results in a decreased signal of geographic structure.

The genetic structure was also analysed using a genetic clustering method without previous assignment of individuals to a geographic origin. The multilocus genotypes of 661 individuals from the whole dataset were assigned to 22 ancestral clusters or populations (Fig.1B). The distribution of these clusters among the sampled field population or LM populations is shown in Fig. 1C. Some clusters showed a narrow correspondence with field populations or geographic regions (Fig.1C). However, other clusters included individuals from different biogeographical provinces, e.g., Clusters 14, 15, and 17 included individuals from AZP and SMO. Clusters including individuals from different biogeographical provinces came from SMO, C and LM populations (Fig. 1C). Interestingly, clusters 3, 11, 12 and 18, only included individuals from C populations. When a similar analysis was done for the 228 multilocus genotypes from W populations, seven ancestral clusters were found (Fig.S2). All individuals from the same location clustered together within a single group with high average ancestry coefficients estimated under admixture model (0.939±0.088 – 0.976±0.039), and each cluster included all individuals from a biogeographical province, except that for AZP individuals were divided into two groups, one including BER-W and the other TUL-W and CER-W. Hence, the results of cluster analyses agree with those from population graphs above in showing strong genetic structure for W populations that is decreased when C populations and LM collections are included in the analysis.

The structure of genetic variation was also examined by the fixation index, *F*_ST_, and actual differentiation, *D*_est_, which showed some very large (global estimates: *F*_ST_  =  0.430 and *D*_est_  =  0.682) and significant (P < 0.05) values between most pairs of field populations, including all pairs of W populations (Table S1). *F*_ST_ and *D*_est_ estimates were correlated (Mantel test, *r*  =  0.578, P< 0.001 for log-transformed values). The lowest divergence value corresponded to populations PEL-W and HUJ-W, 112 km distant. Divergence estimates among some LM populations (XIL-LM, TUL-LM, CER-LM, SLU-LM and EHI-LM) indicated no genetic differentiation (*F*_st_ ranged between 0.005 and 0.032 and *D*_est_ between 0.005 and 0.024). Stepwise AMOVA showed significant latitude x longitude interaction when biogeographical province, level of human management and population were analysed, but no statistical significance was found for latitude or longitude. Population showed the greatest amount of differentiation (Φst|covariates  =  0.4722) followed by biogeographical province (Φst|covariates  =  0.2077) and level of human management (Φst|covariates  =  0.1244). Similar results were obtained when the analysis was restricted to the set of W populations.

### Relationship between geographic and genetic distances at different spatial scales

To determine whether the distribution of genetic variation is structured geographically in the Mexican chiltepin population, isolation by distance (IBD) was analysed using data from W populations. Mantel test showed that genetic distance (*D_A_*) was positively correlated with geographic distance (*r*  =  0.652, *P*< 0.001 for log-transformed data; Fig.S3). Similar results were obtained when *D*_est_ was used (*r*  =  0.526, *P*< 0.001 for log-transformed data). The analysis was repeated for the Eastern and Western populations separately, showing that the positive correlation between geographic and genetic distance was due solely to the Western populations from CPS, CPA and SON (*r*  =  0.882, *P*< 0.006), and not to the Eastern ones (*r*  =  0.198, *P*<0.226).

The spatial genetic structure was also examined at the within-population scale for those W populations in which more than 20 individuals were genotyped. The most likely number of genetic groups was evaluated by cluster analysis, showing genetic structure within five out of eight populations tested, with 3 (HUA-W, BER-W, MOC-W, MAU-W) to 4 (PEL-W) inferred groups for each population (Fig.S4A). The averaged ancestry coefficients of individuals within groups for each population varied from 0.785±0.086 in MOC-W to 0.967±0.014 in MAU-W. The assignment of individual plants to a genetic cluster was unrelated to its spatial position within the population, and Mantel tests showed no correlation between plant distance and genetic distance for populations HUA-W, MOC-W, PEL-W and MAU-W (*P*≥0.197), and a marginally significant correlation for BER-W (*P*  =  0.09), in which plants were sampled along two transects 1 km distant (Fig. S4B).

### Genetic variation in populations from wild and anthropic habitats

To test if human management resulted in a decrease in genetic variation of chiltepin populations the value of *H*_s_, *R*_s_ and *N*_a_ was compared between W, LS and C populations. The mean value and standard error for these indices, estimated for the populations pooled according to the level of human management (W< LS < C), is shown in bold in Table 2. Individuals from HUJ-CHG were not included in these analyses since only four out of the ten individuals from this population grouped with those from W populations of the same region (HUJ-W and PEL-W) by means of cluster analysis. Genetic variation according to any of the three indexes significantly depended on the level of human management (*F*_(2,18)_  =  4.41, *P*  =  0.027; *F*_(2,18)_  =  5.39, *P*  =  0.014; *F*_(2,18)_  =  4.83, *P*  =  0.020 for *H*_s_, *R*_s_ and *N*_a_, respectively), and was lower in the C populations (*t*  =  −2.88, *P*  =  0.009; *t*  =  −3.14, *P*  =  0.005; *t*  =  −2.98, *P*  =  0.007, for *H*_s_, *R*_s_ and *N*_a_, respectively). The significant decrease in the genetic diversity of C populations relative to W ones is particularly clear when *R*_s_ and *N*_a_, are considered, with values being 63% and 68% lower, respectively, for C populations. These results did not vary when the population HUA-CHG was excluded from the analysis. No significant differences were found for any index between W and LSP+LSF populations, regardless of whether the average within population variation or the variation at each locus as repeated measures within populations were considered (*F*_(1,10)_  =  0.00, *P*  =  0.949; *F*_(1,10)_ =  0.04, *P*  =  0.830; *F*_(1,10)_  =  0.09, *P*  =  0.765 for *H*_s_, *R*_s_ and *N*_a_, respectively). Similar analyses with inbreeding coefficient values did not show differences according to the level of human management (*F*_(2,18)_  =  2.19, *P*  =  0.139). Comparable results were obtained when rarefaction analyses that correct for uneven sample sizes were done for the different levels of human management. W populations showed more haplotypes for the more diverse microsatellite (CAMS-885) than the LS+C populations, but LS populations in pastures supported more than twice the number of alleles than any of remaining managed populations (Fig. S5).

**Table 2:** Genetic variation of 34 Mexican populations of *C. annuum* var. *glabriusculum.*

Population^1^	Region^2^	n^3^	*MG* 4	*N*_a_ 5	*PA* 6	*H*_o_ 7	*H*_s_ 8	*R*_s_ 9	*F*_IS_ 10
**Field populations**									
DZI-W	YUC	23	23	11.11±6.60	16(6)	0.570±0.034	0.752±0.066	4.64±1.55	0.246
CHO-CHG	YUC	23	23	6.22±2.39	6(3)	0.506±0.035	0.674±0.053	3.62±0.89	0.253
HUA-W	CPS	21	20	5.11± 2.20	7(4)	0.437±0.036	0.659±0.086	3.45±1.13	0.343
HUA-CHG	CPS	10	1	1.00±0.00	0	0.000±0.000	0.000±0.000	1.00±0.00	-
TLA-W	SMO	23	23	5.56±5.36	3(1)	0.217±0.029	0.468±0.101	2.90±0.00	0.541
TLA-CMC	SMO	16	14	3.00±1.41	2(2)	0.167±0.031	0.356±0.078	2.12±0.00	0.54
PVE-CMC	SMO	22	20	2.78±1.48	0	0.197±0.028	0.316±0.076	2.03±0.83	0.382
TUL-W	AZP	22	22	7.33±6.14	5(3)	0.475±0.035	0.660±0.083	3.77±1.63	0.286
TUL-LSF	AZP	23	21	6.33±3.32	3(1)	0.402±0.034	0.668±0.068	3.67±1.25	0.403
TUL-LSP	AZP	23	23	8.56±8.96	3(1)	0.483±0.035	0.677±0.071	3.88±1.65	0.291
BER-W	AZP	23	22	4.22±4.49	1(1)	0.251±0.030	0.403±0.093	2.34±1.48	0.382
CER-W	AZP	13	13	4.33±2.78	3(1)	0.231±0.039	0.492±0.098	2.92±1.42	0.541
CER-LSP	AZP	20	17	3.11±2.03	1(1)	0.220±0.031	0.345±0.093	2.16±1.42	0.369
CER-CMC	AZP	9	9	2.56±1.88	0	0.099 ±0.033	0.330±0.096	2.12 ±1.19	0.713
LIB-CMC	CPA	14	4	1.44±0.73	0	0.056 ±0.020	0.108±0.067	1.31±0.53	0.497
POT-CHG	CPA	10	4	1.44±0.53	0	0.044±0.022	0.063±0.030	1.22±0.53	0.308
HUJ-W	CPA	11	11	3.44±1.81	0	0.222±0.042	0.508±0.103	2.73±0.53	0.574
HUJ-CHG	CPA	10	8	4.56± 1.24	0	0.244±0.045	0.704±0.035	1.89±0.53	0.167
PEL-W	CPA	23	23	5.44±3.21	1(1)	0.377±0.034	0.571±0.114	3.38±1.59	0.345
ELO-LSP	CPA	23	19	3.67±1.80	1(1)	0.338±0.033	0.458±0.091	2.44±1.59	0.266
SAN-LSP	CPA	23	23	4.78±2.91	5(3)	0.448±0.035	0.518±0.111	2.99±1.41	0.139
SJA-W	SON	23	20	2.78±1.92	1(1)	0.215±0.029	0.362±0.115	2.15±1.14	0.413
MOC-W	SON	23	22	2.44±1.01	0	0.203±0.028	0.411±0.101	2.11±0.86	0.512
MAZ-LSF	SON	15	15	2.22±1.20	0	0.106±0.027	0.319±0.092	1.89±0.86	0.675
MAU-W	SON	23	9	2.44±1.88	1(1)	0.050±0.015	0.158±0.063	1.60±0.74	0.689
TEM-CMC	SON	20	19	3.00±1.50	1(1)	0.168±0.028	0.429±0.089	2.34±0.74	0.614
HER-CMC	SON	23	17	2.78±1.92	0	0.069±0.018	0.319±0.113	2.07±1.19	0.787
**Pooled**	**W**	**228**	**208**	**22.78±20.27**	**48(8)**	**0.299±0.010**	**0.796±0.053**	**19.91±16.10**	**0.624**
**Pooled**	**LS**	**127**	**118**	**15.67±13.77**	**18(4)**	**0.350±0.014**	**0.746±0.086**	**15.61±13.77**	**0.531**
**Pooled**	**C**	**157**	**119**	**14.00±8.47**	**10(4)**	**0.179±0.010**	**0.793±0.049**	**13.52±7.87**	**0.775**
**Pooled**		**512**	**445**	**27.00±26.56**		**0.275±0.006**	**0.815±0.056**	**8.26±2.96**	
**Local markets**									
XIL-LM	SMO	23	23	3.67±1.22	0	0.188±0.027	0.471±0.054	2.58±0.73	0.605
TUL-LM	AZP	23	23	4.67±2.55	2(1)	0.273±0.031	0.504±0.067	2.81±1.08	0.465
TOL-LM	AZP	15	15	4.00±2.55	4(2)	0.326±0.040	0.527±0.084	2.83±1.20	0.39
SLU-LM	AZP	23	23	4.89±2.47	0	0.373±0.034	0.576±0.060	3.07±1.01	0.358
CER-LM	AZP	19	19	4.78±2.64	1(1)	0.234±0.032	0.526±0.066	2.91±1.01	0.562
EHI-LM	CPA	23	23	5.33±2.65	0	0.211±0.029	0.569±0.068	3.15±0.53	0.635
BAT-LM	SON	23	18	2.33±1.50	0	0.102±0.021	0.281±0.096	1.89±1.41	0.642
**Pooled**		**149**	**144**	**11.22±7.22**	**9(3)**	**0.239±0.011**	**0.684±0.044**	**6.47±2.75**	**0.651**
**TOTAL**		**661**	**589**	**28.00±28.46**		**0.267±0.005**	**0.817±0.048**	**5.06±1.35**	

1 Populations are named with the three first letters of the name of the nearest village, plus a code indicating the habitat: W =  wild, LSP =  Let standing, pasture; LSF =  Let standing, living fence; CHG =  Cultivated, home garden; CMC =  Cultivated, monoculture, LM =  Seeds from fruits purchased at local markets.

2 YUC: Yucatan; CPS: Costa del Pacífico Sur; SMO: Sierra Madre Oriental; AZP: Altiplano Zacatecano-Potosino; CPA: Costa del Pacífico; SON: Sonora.

3 Sample size.

4 Number of multilocus genotypes.

5 Mean number of alleles±standard error.

6 Number of private alleles. The number of loci containing private alleles is indicated in parenthesis.

7 Mean observed heterozygosity±standard error.

8 Mean unbiased expected heterozygosity±standard error.

9 Mean allelic richness±standard error.

10 Inbreeding coefficient.

### Demographic history of wild populations

Genetic data analysed with MSVAR1.3 (Table 3) show demographic declines in all W populations from an 18 fold decline in the population formed by HUJ-W plus PEL-W to a 373 fold decline in BER-W, with most population declines being around 35 to 79 fold. Ages of these declines vary from 2710 years in DZI-W to 43251 years in TLA-W, with other values between 6000 to 17000 years. Mutation rate estimates, which were independently estimated for each population, were highly consistent and very close to 2.8×10^-4^.

**Table 3:** Demographic history of wild *C. annuum var. glabriusculum* populations or groups of populations.

Population^1^	*N*_0_ 2	*N*_1_ 3	*t*_a_ 4	*µ* 5	*N*_1_/ *N*_0_
DZl-W	1524 (167–13274)	52723(9863–263027)	2710(242–26122)	2.6 (0.90–8.20)	34.59
HUA-W	794(61–6516)	29444(4667–173780)	6166(283–114025)	2.6 (0.89–8.18)	37.08
TLA-W	1047(157–6223)	55463(3296–781628)	43251 (2046–756833)	2.7 (0.92–8.34)	52.97
SON-W	392(45–2805)	30832(2234–400667)	8299(422–204174)	2.6 (0.89–7.96)	78.65
TUL-W+CER-W	1862(286–9441)	66988 (10399–426580)	17061 (1581–171396)	2.8 (0.95–8.67)	35.97
BER-W	187 (15–1315)	69823(7178–622300)	6012 (485–61801)	2.6 (0.87–8.00)	373.38
HUJ-W+PEL-W	1986(191–15776)	36224(5848–227510)	7870(301–162930)	2.7 (0.91–8.30)	18.24
All (wild)	6266 (1321–25177)	93541 (23605–389045)	10740 (2455–47936)	2.8 (0.91–8.30)	14.92

1 Populations or groups of populations are named with the three first letters of the name of the nearest village or the code of the subregion, respectively, plus the code of the habitat, W.

2 Current effective number of individuals.

3 Number of individuals at the time where the expansion/decline began.

4 Number of years since the beginning of the expansion/decline.

5 Mutation rate (x 10^4^).

Credibility intervals are shown between parenthesis for all parameters.

## Discussion

Here we analyse the possible impact of pre-domestication human management on the genetic variation and population structure of the wild pepper or chiltepin. For this we compared, in different biogeographic provinces of Mexico, neighbouring field populations with different degrees of human management: wild, let standing and cultivated populations (*sensu* Casas *et al*. [25]). Genetic variation was analysed using nine nuclear microsatellite markers, which were found to be highly polymorphic. Genetic diversity was highest for the YUC populations, which share many alleles with the other populations, and decreased towards the North and West limit of the chiltepin geographical range, being lowest in SON populations. These results agree with reports of the Yucatan peninsula as a centre of diversity of several important Mesoamerican domesticates and their wild relatives [22], [45]. A decline in within-population genetic diversity towards range periphery is predicted to be due to smaller effective population sizes and increased geographical isolation. This prediction is true in about 65% of analysed plant species [46]. Our analyses provide evidence of a strong geographic structure, and isolation by distance, for W populations. This is in agreement with previous reports based on isozyme or RAPDs markers of domesticated, semi-domesticated and wild accessions of *C. annuum* from Mexico [18], [24], [47]. Other reports, however, failed to detect geographic structure of wild pepper populations, which can probably be explained by their more limited geographic scope [23]. Observed geographic structure could be explained by different factors, including the mountain ranges acting as barriers for chiltepin dispersion, the discontinuous distribution of the plant, or the limited ranges of activity of seed-dispersing birds [15], [18]. On the other hand, dispersal distances of chiltepin seeds by birds [48], [49] and foraging ranges of pollinator bees [50], could explain the lack of spatial structure at the within-population scale, as most analysed populations only extended for a few hundred square meters (not shown).

The strong genetic structure shown in W populations was not affected by the inclusion in the analyses of LS ones, but was not as clear when C and LM populations were included, as many C or LM populations did not cluster with neighbouring W or LS populations. Structure-based analyses of genetic clustering also led to similar results. An important conclusion of these results is that material purchased at local markets often does not come from local populations, issuing a warning against its use in analyses of genetic variation and structure of plant populations, or as material for *ex situ* preservation of genetic diversity [51], as has been done in the past [22], [47]. Most seeds for small-scale monocultures derive from SMO populations, which might be the origin of chiltepin cultivation. Results also suggest that there is an active translocation of fruits for their commerce. Loss of genetic structure in C populations due to long-distance translocation and genotype mixing has also been described for other incipient domesticates, as well as for crops under traditional agricultural systems in Mexico [51]–[53]. Interestingly, LS and C populations act as bridges for gene flow among W ones (compare Fig. 2 and Fig. S1A), thus representing a threat of erosion of population structure, and loss of local adaptations. However, given the fragmentation of W habitats, human-mediated dispersal could avoid local extinction in some areas. Therefore, the effects of this breakdown of isolation on the long term genetic diversity and survival of populations could be relevant for future conservation policies, and should be studied further.

While tolerance or protection of plants in some anthropic habitats such as pastures did not result in a large loss of genetic diversity, in others, such as live fences, the levels of variation were lower (Fig. S5). This observation is at odds with reports for other plant species undergoing domestication in Mexico, for which favourable phenotypes are selected in managed and in LS populations [6], [54]–[56]. Our results are compatible with a lack of selection on LS chiltepin plants, which were phenotypically undistinguishable from their sympatric W counterparts (our observation). However, in the C populations there was a significant decrease in genetic variation relative to neighbouring W or LS populations. A common trend in plant domestication is a loss of genetic variation [3]–[5], [57] that varies largely according to the plant species and the set of wild and domesticated accessions analysed [5], [8], [45], [51], [53], [57]–[59]. It has been proposed that domestication of *C. annuum* has resulted in no or a low (around 10%) decrease of variation [22]–[24]. Our results indicate a reduction of genetic variation up to 50%, which could be attributed to our using a larger chiltepin sample and different molecular markers. The 30–40% reduction in mean number of alleles and in allelic richness detected for C populations is quite striking, and could be due to strong population bottlenecks associated to a low number of cultivation origins. Artificial and directional selection has probably not yet played an important role, as chiltepin cultivation has not been paralleled by domestication. Chiltepin plants from C populations, or derived from commercial fruit batches, do not differ from W population plants in any trait associated with the domestication syndrome in pepper [21]. The only obvious trait associated with incipient domestication in cultivated chiltepin is an increase in germination rate, which is highly variable for W populations (2–50 % for most populations, [60], [61] and our unpublished data), a trait which was universally held as the major barrier to cultivation by all our local informants. Germination was about 70% in C or LM populations (our unpublished data). In spite of the important reduction of genetic variation, C and LM populations include genetic diversity not detected in W or LS populations, as shown by the presence of private alleles, and of specific genotypes. This may reflect the limitations of our wild-population sample but, more significantly, is evidence that traditional managed habitats may be relevant reservoirs of genetic variation, particularly when native W populations are declining [53]. We were able to detect this recent demographic decline in W populations (Table 3), which varied for different populations. This suggests the heterogeneous effects of habitat fragmentation due to climate fluctuations during glacial and interglacial periods, or due to anthropogenic causes after the human settling of America. The broad time span detected indicates that the population decline is multifactorial and should be studied in detail for each particular case. For example, the population decline in DZI-W roughly coincides with the onset of mayan civilization in the Yucatan peninsula.

Wild relatives of crops, such as chiltepin, represent a potential source of genetic diversity to cope with new needs in crop improvement, and their conservation has been emphasized as an important objective requiring international collaboration [62], [63]. The conservation of plant genetic resources has historically focused on *ex situ* preservation of crop varieties or landraces. The fraction of wild accessions in *ex situ* collections is still low, and large gaps in the species diversity most often remain to be covered [63]–[66]. A complementary strategy is *in situ* conservation, as it maintains the ability of the species to evolve in native conditions and, therefore, its potential genetic variation. *In situ* conservation has only been addressed rigorously over the last decade, when the growing rate of species extinction and its causes were identified. *In situ* conservation of chiltepin would require selecting areas including wild populations in the various geographical regions where individual clusters of genetic diversity have been identified, as well as areas of incipient domestication, which maintain the architecture of genetic variation. Due to the strong spatial genetic structure of chiltepin revealed here, to develop such a conservation programme would require a more detailed analysis of the genetic structure of wild chiltepin populations, with a denser coverage of its area of distribution than was attempted in this work. These analyses should not be delayed as population decline and fast alteration of native habitats may be a factor accelerating chiltepin domestication.

## Supporting Information

Figure S1 **Population graphs of A. wild, and B. let standing/cultivated populations of chiltepin.** See Figure 2 for population notation. The origin in six biogreographic provinces in Mexico of the wild populations within each cluster is shown with the following symbols. §, YUC  =  Yucatan, $, SMO  =  Sierra Madre Oriental, *, AZP  =  Altiplano Zacatecano Potosino, ¥, CPS  =  Costa del Pacífico Sur, ^¤^, CPA  =  Costa del Pacifico, #, SON  =  Sonora, £, SIN  =  Sinaloa.
(PDF)

Figure S2 **Genetic structure of wild** ***C. annuum*** **var.** ***glabriusculum*** **populations.** Two hundred and twenty eight genotyped individuals clustered into 7 groups. Each individual is represented by a vertical bar, which is divided into 7 coloured fractions representing the estimated portion of its genome that assigns the individual to each of 7 clusters. Black lines separate different clusters. Biogeographical provinces are indicated at the top of the chart.
(PDF)

Figure S3 **Correlation between geographic and genetic distance in eleven wild populations of** ***C. annuum*** **var.** ***glabriusculum*** **.** Log-transformed data are presented.
(PDF)

Figure S4 **Local spatial structure in wild populations of** ***C. annuum*** **var.** ***glabriusculum*** **.** (A) Within-population substructure was found in five out of eight wild populations tested. To facilitate comparisons, individuals are arranged according to the location within the sampled transects. Each individual is represented by a thin vertical bar which is divided into *K* coloured fractions representing the estimated portion of its genome that assigns the individual to each *K* cluster. Thin bars below grouped individuals according to their aggregation in the field and bold bars below represent the length of transects. (B) Correlation between geographic and genetic distances of individuals for populations BER-W and MOC-W. Log-transformed data are presented.
(PDF)

Figure S5 **Rarefaction analyses for different habitats in** ***C. annuum*** **var.** ***glabriusculum*** **and the most variable microsatellite, CAMS-885.**
(PDF)

Table S1 **Values of the fixation index** ***F_ST_*** **(above the diagonal) and and** ***D*** **_est_ distance values (below the diagonal) between pairs of Mexican populations of** ***Capsicum annuum*** **var.** ***glabriusculum*** **from Mexico.**
(DOCX)

## References

[1] Power AG (2010). Ecosystem services and agriculture: trade-offs and synergies.. Phil Trans Royal Soc B.

[2] Rands MRW, Adams WM, Bennun L, Butchart SHM, Clements A (2010). Biodiversity Conservation: Challenges Beyond 2010.. Science.

[3] Doebley JF, Gaut BS, Smith BD (2006). The molecular genetics of crop domestication.. Cell.

[4] Gross BL, Olsen KM (2010). Genetic perspectives on crop domestication.. Trends Plant Sci.

[5] Tang H, Sezen U, Paterson AH (2010). Domestication and plant genomes.. Curr Op Plant Biol.

[6] Blanckaert I, Vancraynest K, Swennen RL, Espinosa-García FJ, Piñero D (2007). Non-crop resources and the role of indigenous knowledge in semi-arid production of Mexico.. Agr Ecosyst Environ.

[7] Casas A, Valiente-Banuet A, Viveros JL, Caballero J, Cortés L (2001). Plant resources of the Tehuacán-Ciucatlán Valley, Mexico.. Econ Bot.

[8] Otero-Arnaiz A, Casas A, Hamrick JL, Cruse-Sanders J (2005). Genetic variation and evolution of *Polaskia chichipe* (Cactaceae) under domestication in the Tehuacán Valley, central Mexico.. Mol Ecol.

[9] Pickersgill B (2007). Domestication of plants in the Americas: Insights from mendelian and molecular genetics.. Annals Bot.

[10] Zeder MA (2006). Central questions in the domestication of plants and animals.. Evol Anthropol.

[11] DArcy WG, Eshbaugh WH (1974). New World peppers (*Capsicum*, Solanaceae) north of Colombia: a resume.. Baileya.

[12] Pickersgill B (1971). Relationship between weedy and cultivated forms in some species of chilli peppers (genus *Capsicum*).. Evolution.

[13] Pickersgill B (1997). Genetic resources and breeding of *Capsicum* spp.. Euphytica.

[14] Hernández-Verdugo S, Dávila-Aranda P, Oyama K (1999). Síntesis del conocimiento taxonómico, origen y domesticación del género *Capsicum*.. Bol Soc Bot Mex.

[15] Tewksbury JJ, Nabhan GP, Norman D, Suzan H, Tuxill J (1999). *In situ* conservation of wild chiles and their biotic associates.. Conserv Biol.

[16] Hernández-Verdugo S, Guevara-González RG, Rivera-Bustamante S, Oyama K (2001). Screening wild plants of *Capsicum annuum* for resistance to Pepper Huasteco Virus: Presence of viral DNA and differentiation among populations.. Euphytica.

[17] Smith CE, Byes DS (1967). Plant remains.. The prehistory of the Tehuacan Valley, vol.1 Environment and subsistence.

[18] Votava EJ, Nabham GP, Bosland PW (2002). Genetic diversity and similarity revealed via molecular analysis among and within an *in situ* population and *ex situ* accessions of chiltepin (*Capsicum annuum* var. *glabriusculum*).. Conserv Genet.

[19] Nabhan GP (1990). Conservationists and Forest Service join forces to save wild chiles.. Diversity.

[20] Rodríguez del Bosque LA, Pozo-Campodónico O, Ramírez-Meraz M, Silva-Cavazos FJ (2002). Effect of shading on growth and yield of ten accessions of piquin pepper (*Capsicum annuum* var..

[21] Paran I, van der Knaap E (2007). Genetic and molecular regulation of fruit and plant domestication traits in tomato and pepper.. J Exp Bot.

[22] Aguilar-Meléndez A, Morell PL, Roose ML, Kim SC (2009). Genetic diversity and structure in semiwild and domesticated chiles (*Capsicum annuum*; Solanaceae) from Mexico.. Am J Bot.

[23] Hernández-Verdugo S, Luna-Reyes R, Oyama K (2001). Genetic structure and differentiation of wild and domesticated populations of *Capsicum annuum* (Solanaceae) from Mexico.. Plant Syst Evol.

[24] Oyama K, Hernández-Verdugo S, Sánchez C, González-Rodríguez A, Sánchez-Peña P (2006). Genetic structure of wild and domesticated populations of *Capsicum annuum* (Solanaceae) from northwestern Mexico analyzed by RAPDs.. Genet Res Crop Evol.

[25] Casas A, Otero-Arnaiz A, Pérez-Negrón E, Valiente-Banuet A (2007). *In situ* management and domestication of plants in Mesoamerica.. Ann Bot.

[26] Comisión Nacional para el Conocimiento y Uso de la Biodiversidad(CONABIO) (1997). Provincias biogeográficas de México..

[27] Minaniyama Y, Tsuro M, Hirai M (2006). An SSR-based linkage map of *Capsicum annuum*.. Mol Breed.

[28] Nei M (1987). Molecular Evolutionary Genetics..

[29] El Mousadik A, Petit RJ (1996). High level of genetic differentiation for allelic richness among populations of the argan tree [*Argania spinosa* (L.) Skeels] endemic to Morocco.. Theor Appl Genet.

[30] Goudet J (2001). FSTAT, a program to estimate and test gene diversities and fixation indices (version 2.9.3).. http://www.unil.ch/izea/softwares.

[31] Peakall R, Smouse PE (2006). GENALEX 6: Genetic analysis in Excel. Population genetic software for teaching and research.. Mol Ecol Notes.

[32] Weir BS, Cockerham CC (1984). Estimating F-statistics for the analysis of population structure.. Evolution.

[33] Crawford NG (2010). SMOGD: software for the measurement of genetic diversity.. Mol Ecol Res.

[34] Jost L (2008). *G*
_ST_ does not measure genetic differentiation.. Mol Ecol.

[35] Dyer RJ (2009). Genetic Studio: A suite of programs for the spatial analysis of genetic marker data.. Mol Ecol Res.

[36] Dyer RJ, Westfall RD, Sork VL, Smouse PE (2004). Two-generation analysis of pollen flow across a landscape V: a stepwise approach for extracting factors contributing to pollen structure.. Heredity.

[37] Dyer, RJ, Nason JD (2004). Population graphs: The graph-theoretic shape of genetic structure.. Mol Ecol.

[38] Falush D, Stephens M, Pritchard JK (2003). Inference of population structure using multilocus genotype data: linked loci and correlated allele frequencies.. Genetics.

[39] Pritchard JK, Stephens M, Donnelly P (2000). Inference of population structure using multilocus genotype data.. Genetics.

[40] Jensen JL, Bohonak AJ, Kelley ST (2005). Isolation by distance, web service.. BMC Genet.

[41] Beaumont MA (1999). Detecting population expansion and decline using microsatellites.. Genetics.

[42] Beaumont MA (2004). msvar1.3 update.. http://www.rubic.rdg.ac.uk/mab/stuff/.

[43] Storz JF, Beaumont MA (2002). Testing for genetic evidence of population expansion and contraction: an empirical analysis of microsatellite DNA variation using a hierarchical Bayesian model.. Evolution.

[44] Drummond AJ, Rambaut A (2007). BEAST: Bayesian evolutionary analysis by sampling trees.. BMC Evol Biol.

[45] Martínez-Castillo J, Colunga-GarcíaMarín P, Zizumbo D (2008). Genetic erosion and in situ conservation of Lima bean (*Phaseolus lunatus* L.) landraces in its Mesoamerican diversity center.. Genet Res Crop Evol.

[46] Eckert CG, Samis KE, Lougheed C (2008). Genetic variation across species geographical ranges: the central-marginal hypothesis and beyond.. Mol Ecol.

[47] Loaiza-Figueroa F, Ritland K, Laborde-Cancino JA, Tanksley SD (1989). Patterns of genetic variation of the genus *Capsicum* (*Solanaceae*) in Mexico.. Plant Syst Evol.

[48] Carlo TA, Tewksbury JJ, Martínez del Río C (2009). A new method to track seed dispersal and recruitment using ^15^N isotope enrichment.. Ecology.

[49] Tewksbury JJ, Levey D, Huizinga M, Haak DC, Traveset A (2008). Costs and benefits of capsaicin-mediated control of gut retention in dispersers of wild chilies.. Ecology.

[50] Greenleaf SS, Williams NM, Winfree R, Kremen C (2007). Bee foraging ranges and their relationship to body size.. Oecologia.

[51] Zizumbo-Villarreal D, Colunga-GarcíaMarín P, Payro de la Cruz E, Delgado-Valerio P, Gepts P (2005). Population structure and evolutionary dynamics of wild-weedy-domesticated complexes of common bean in a Mesoamerican region.. Crop Sci.

[52] Louette DA, Charrier A, Berthaud J (1997). In situ conservation of maize in Mexico: Genetic diversity and maize seed management in a traditional community.. Econ Bot.

[53] Miller A, Schaal B (2005). Domestication of a Mesoamerican cultivated fruit tree, *Spondias purpurea*.. Proc Natl Acad Sci USA.

[54] Carmona A, Casas A (2005). Management, phenotypic patterns and domestication of *Polaskia chichipe* (Cactaceae) in the Tehuacán Valley, Central Mexico.. J Arid Environ.

[55] Casas A, Caballero J, Valiente-Banuet A, Soriano JA, Dávila P (1999). Morphological variation and the process of domestication of *Stenocereus stellatus* (Cactaceae) in central Mexico.. Am J Bot.

[56] Otero-Arnaiz A, Casas A, Bartolo C, Pérez-Negrón E, Valiente-Banuet A (2003). Evolution of *Polaskia chichipe* (Cactaceae) under domestication in the Tehuacán Valley, central Mexico: reproductive biology.. Am J Bot.

[57] Buckler ES, Thornsberry JM, Kresovich S (2001). Molecular diversity, structure and domestication of grasses.. Genet Res.

[58] Colunga-GarcíaMarín P, Coello-Coello J, Eguiarte LE, Piñero D (1999). Isozymatic variation and phylogenetic relationships between henequén (*Agave fourcroydes*) and its wild ancestor *A. angustifolia* (Agaveaceae).. Am J Bot.

[59] Sonnante G, Stockton T, Nodari RO, Becerra-Velásquez VL, Gepts P (1994). Evolution of genetic diversity during the domestication of common-bean (*Phaseolus vulgaris* L.).. Theor Appl Genet.

[60] Hernández-Verdugo S, Oyama K, Vázquez-Yanes C (2001). Differentiation in seed germination among populations of *Capsicum annuum* along a latitudinal gradient in Mexico.. Plant Ecol.

[61] Ramírez-Meraz M, Pozo-Campodónico O, Rodríguez del Bosque LA, Medina-Martínez T, Villalón M (2002). Production technology for piquen pepper *(Capsicum annuum var. aviculare).*.

[62] Maxted N, Kell SP (2009). Establishment of a global network for the in situ conservation of crop wild relatives: status and needs.. FAO consultancy report, FAO, Rome,.

[63] Khoury C, Laliberté B, Guarino L (2010). Trends in *ex situ* conservation of plant genetic resources: a review of global crop and regional conservation strategies.. Genet Res Crop Evol.

[64] Damania AB (2008). History, achievements and current status of genetic resources conservation.. Agron J.

[65] Maxted N, Ford-Lloyd BV, Kell SP, Iriondo JM, Dulloo ME (2008). Crop wild relatives conservation and use..

[66] Meilleur BA, Hodgkin T (2004). *In situ* conservation of crop wild relatives: status and trends.. Biodivers Conserv.

