# Supplementary figures and images for: Impact of Human Management on the Genetic Variation of Wild Pepper, Capsicum annuum var. glabriusculum
Source: PLoS One. 2011 Dec 6;6(12):e28715. doi: 10.1371/journal.pone.0028715 (PMC3232243; doi:10.1371/journal.pone.0028715)

SFig1a

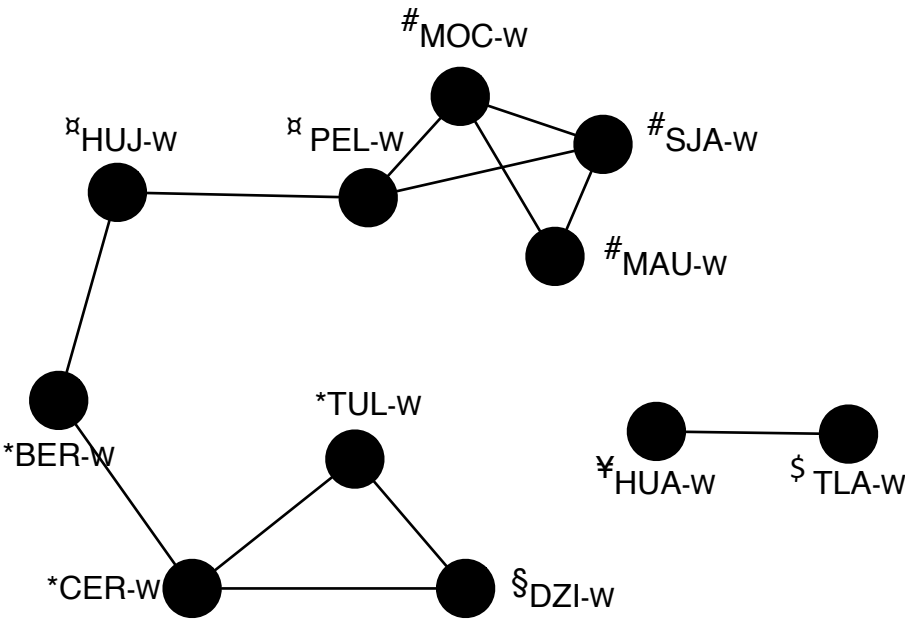

SFig1b

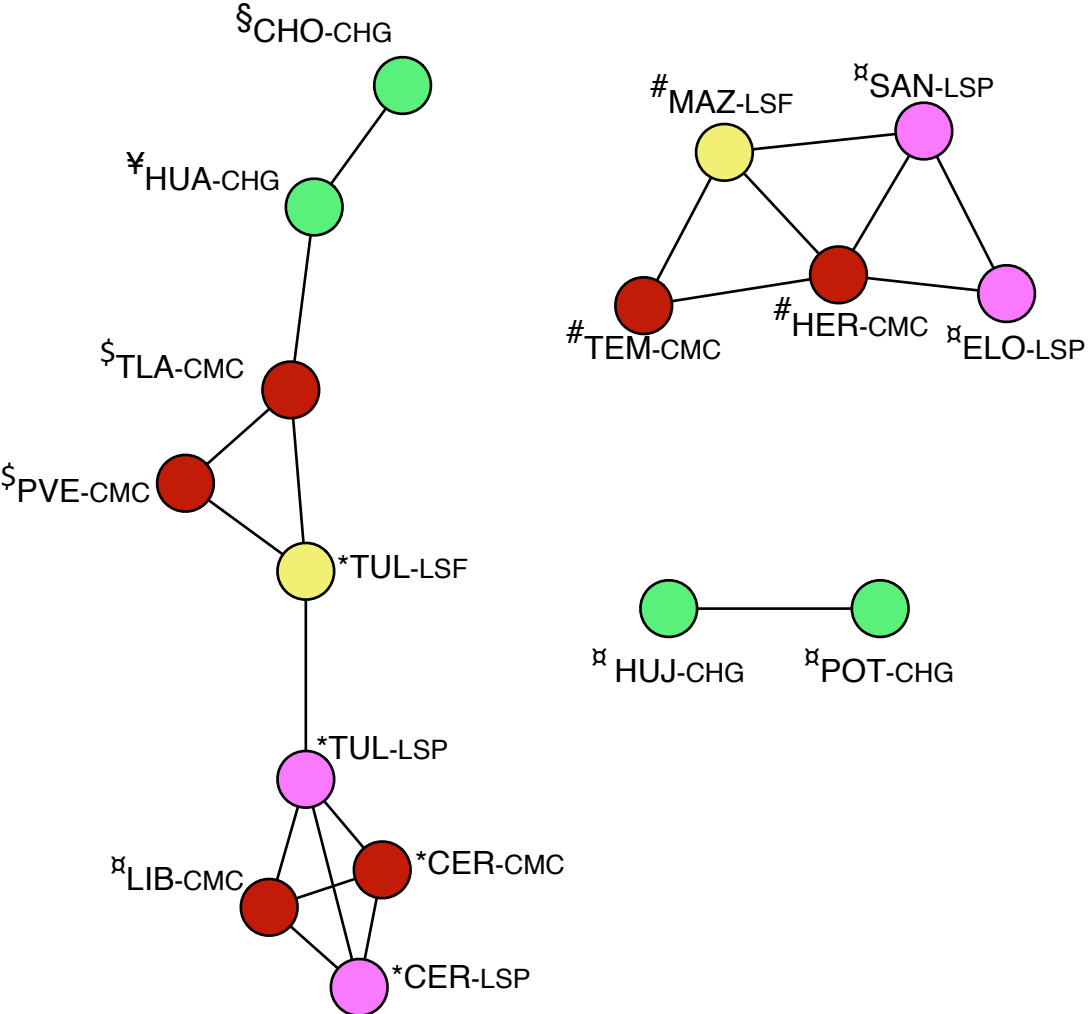

Supplement: Figure S1 — Population graphs of A. wild, and B. let standing/cultivated populations of chiltepin. See Figure 2 for population notation. The origin in six biogreographic provinces in Mexico of the wild populations within each cluster is shown with the following symbols. §, YUC = Yucatan, $, SMO = Sierra Madre Oriental, *, AZP = Altiplano Zacatecano Potosino, ¥, CPS = Costa del Pacífico Sur, ¤, CPA = Costa del Pacifico, #, SON = Sonora, £, SIN = Sinaloa. (PDF) [file pone.0028715.s001.pdf]

**SFig.3**

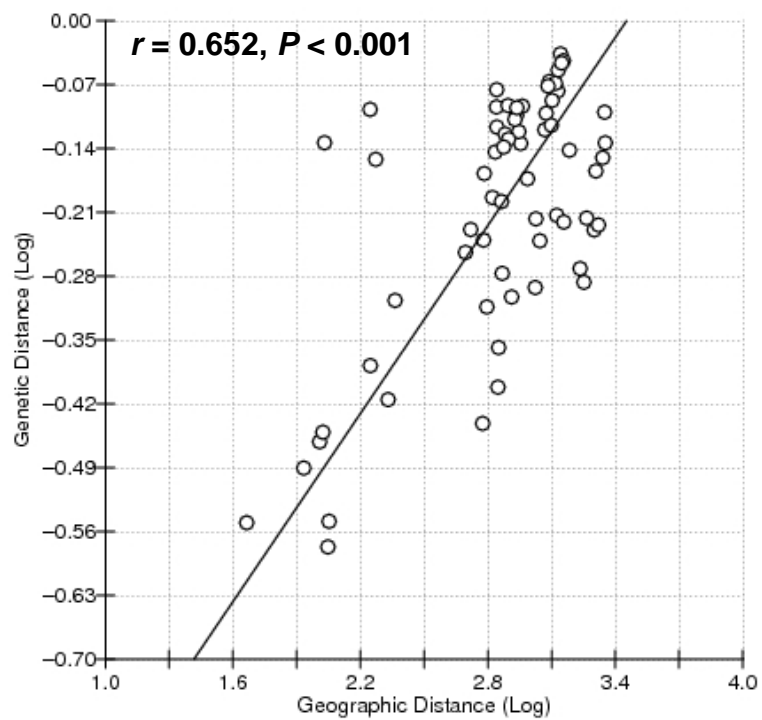

Supplement: Figure S3 — Correlation between geographic and genetic distance in eleven wild populations of C. annuum var. glabriusculum . Log-transformed data are presented. (PDF) [file pone.0028715.s003.pdf]

# SFig 4

A

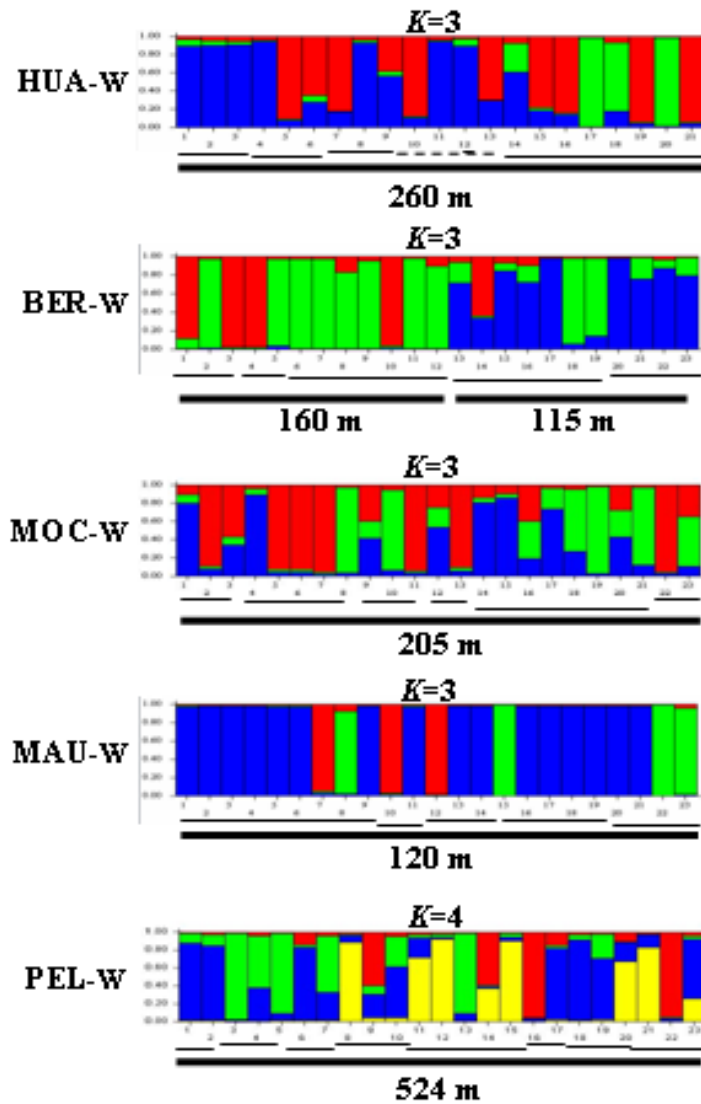

B

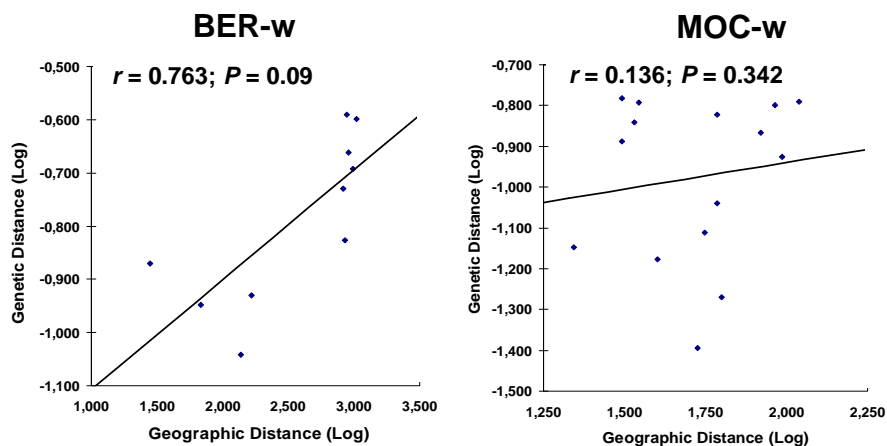

Supplement: Figure S4 — Local spatial structure in wild populations of C. annuum var. glabriusculum . (A) Within-population substructure was found in five out of eight wild populations tested. To facilitate comparisons, individuals are arranged according to the location within the sampled transects. Each individual is represented by a thin vertical bar which is divided into K coloured fractions representing the estimated portion of its genome that assigns the individual to each K cluster. Thin bars below grouped individuals according to their aggregation in the field and bold bars below represent the length of transects. (B) Correlation between geographic and genetic distances of individuals for populations BER-W and MOC-W. Log-transformed data are presented. (PDF) [file pone.0028715.s004.pdf]

**SFig. 5**

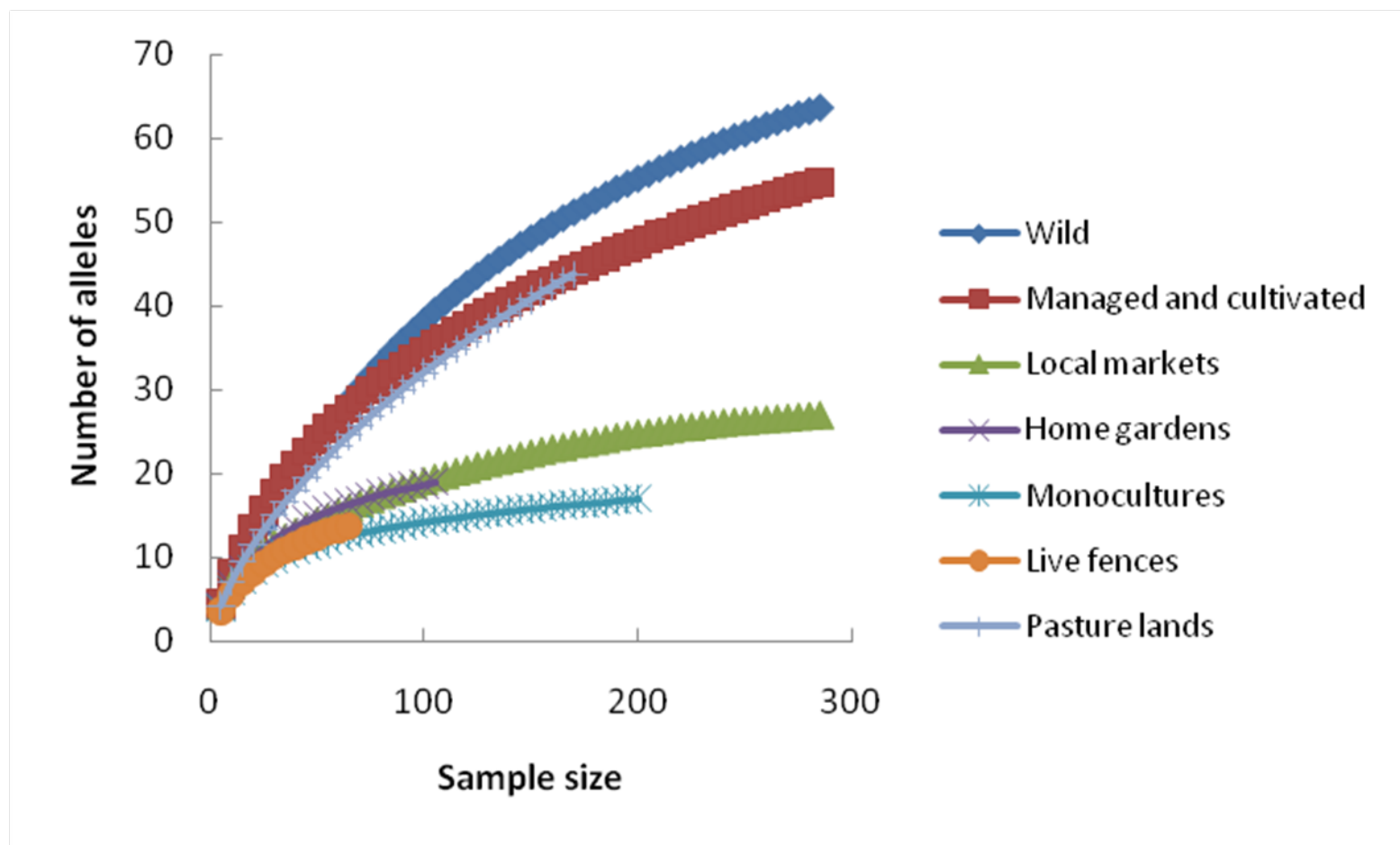

Supplement: Figure S5 — Rarefaction analyses for different habitats in C. annuum var. glabriusculum and the most variable microsatellite, CAMS-885. (PDF) [file pone.0028715.s005.pdf]
