# Supplementary material for: Impact of Human Management on the Genetic Variation of Wild Pepper, Capsicum annuum var. glabriusculum
Source: PLoS One. 2011 Dec 6;6(12):e28715. doi: 10.1371/journal.pone.0028715 (PMC3232243; doi:10.1371/journal.pone.0028715)
Supplement: Table S1 — Values of the fixation index FST (above the diagonal) and and D est distance values (below the diagonal) between pairs of Mexican populations of Capsicum annuum var. glabriusculum from Mexico. (DOCX) [file pone.0028715.s006.docx]

|  | DZIw | CHOag | HUAw | HUAag | TLAw | TLAam | PVEam | XILs | SLUs | TULs | EHIs | CERs | TULw | TULal | TULap | BERw | TOLs | CERw | CERap | CERam | LIBam | POTag | HUJw | HUJag | PELw | ELOap | SANap | SJAw | EAGam | BATs | MAUw | TEMam | MOCw | MAZal |
| --- | --- | --- | --- | --- | --- | --- | --- | --- | --- | --- | --- | --- | --- | --- | --- | --- | --- | --- | --- | --- | --- | --- | --- | --- | --- | --- | --- | --- | --- | --- | --- | --- | --- | --- |
| DZIw | 0,000 | 0,201 | 0,260 | 0,498 | 0,341 | 0,374 | 0,421 | 0,324 | 0,265 | 0,315 | 0,264 | 0,286 | 0,144 | 0,159 | 0,147 | 0,337 | 0,244 | 0,249 | 0,346 | 0,308 | 0,429 | 0,369 | 0,181 | 0,251 | 0,189 | 0,283 | 0,229 | 0,361 | 0,379 | 0,405 | 0,482 | 0,306 | 0,336 | 0,361 |
| CHOag | 0.560 | 0,000 | 0,254 | 0,526 | 0,314 | 0,363 | 0,412 | 0,290 | 0,218 | 0,259 | 0,240 | 0,262 | 0,242 | 0,257 | 0,226 | 0,360 | 0,315 | 0,346 | 0,440 | 0,420 | 0,530 | 0,511 | 0,337 | 0,392 | 0,333 | 0,402 | 0,365 | 0,447 | 0,465 | 0,500 | 0,553 | 0,424 | 0,428 | 0,442 |
| HUAw | 0.820 | 0.585 | 0,000 | 0,530 | 0,380 | 0,433 | 0,449 | 0,385 | 0,324 | 0,359 | 0,326 | 0,351 | 0,302 | 0,295 | 0,290 | 0,440 | 0,363 | 0,368 | 0,451 | 0,415 | 0,569 | 0,560 | 0,269 | 0,291 | 0,248 | 0,329 | 0,310 | 0,344 | 0,369 | 0,415 | 0,487 | 0,341 | 0,351 | 0,352 |
| HUAag | 0.827 | 0.740 | 0.692 | 0,000 | 0,624 | 0,736 | 0,731 | 0,626 | 0,556 | 0,597 | 0,552 | 0,588 | 0,572 | 0,548 | 0,552 | 0,714 | 0,654 | 0,695 | 0,756 | 0,820 | 0,928 | 0,960 | 0,722 | 0,900 | 0,620 | 0,701 | 0,663 | 0,750 | 0,783 | 0,800 | 0,890 | 0,697 | 0,714 | 0,800 |
| TLAw | 0.776 | 0.537 | 0.771 | 0.596 | 0,000 | 0,076 | 0,191 | 0,074 | 0,081 | 0,094 | 0,089 | 0,087 | 0,391 | 0,372 | 0,370 | 0,499 | 0,426 | 0,466 | 0,546 | 0,534 | 0,610 | 0,549 | 0,469 | 0,538 | 0,451 | 0,499 | 0,471 | 0,563 | 0,582 | 0,603 | 0,678 | 0,496 | 0,545 | 0,563 |
| TLAam | 0.780 | 0.546 | 0.772 | 0.603 | 0.041 | 0,000 | 0,341 | 0,163 | 0,171 | 0,190 | 0,162 | 0,176 | 0,441 | 0,425 | 0,423 | 0,552 | 0,487 | 0,529 | 0,611 | 0,613 | 0,720 | 0,674 | 0,511 | 0,631 | 0,489 | 0,538 | 0,522 | 0,611 | 0,638 | 0,661 | 0,738 | 0,553 | 0,591 | 0,622 |
| PVEam | 0.837 | 0.580 | 0.697 | 0.566 | 0.114 | 0.145 | 0,000 | 0,209 | 0,192 | 0,195 | 0,155 | 0,209 | 0,479 | 0,456 | 0,450 | 0,601 | 0,524 | 0,558 | 0,625 | 0,634 | 0,697 | 0,685 | 0,579 | 0,645 | 0,539 | 0,601 | 0,557 | 0,649 | 0,666 | 0,686 | 0,759 | 0,582 | 0,626 | 0,653 |
| XILs | 0.759 | 0.456 | 0.782 | 0.604 | 0.039 | 0.064 | 0.093 | 0,000 | 0,017† | 0,005† | 0,032† | 0,019† | 0,373 | 0,359 | 0,353 | 0,478 | 0,415 | 0,450 | 0,534 | 0,525 | 0,563 | 0,561 | 0,461 | 0,522 | 0,447 | 0,499 | 0,457 | 0,560 | 0,576 | 0,599 | 0,674 | 0,497 | 0,543 | 0,553 |
| SLUs | 0.705 | 0.414 | 0.752 | 0.603 | 0.062 | 0.111 | 0.119 | 0.011† | 0,000 | 0,012† | -0,003† | 0,010† | 0,305 | 0,295 | 0,287 | 0,407 | 0,339 | 0,382 | 0,471 | 0,460 | 0,511 | 0,494 | 0,403 | 0,454 | 0,394 | 0,449 | 0,402 | 0,506 | 0,525 | 0,546 | 0,621 | 0,442 | 0,483 | 0,497 |
| TULs | 0.793 | 0.425 | 0.755 | 0.587 | 0.058 | 0.100 | 0.082 | 0.011† | 0.010† | 0,000 | 0,005† | 0,011† | 0,355 | 0,343 | 0,335 | 0,445 | 0,382 | 0,427 | 0,512 | 0,505 | 0,559 | 0,562 | 0,446 | 0,496 | 0,436 | 0,484 | 0,451 | 0,544 | 0,566 | 0,585 | 0,658 | 0,479 | 0,532 | 0,537 |
| EHIs | 0.702 | 0.416 | 0.744 | 0.589 | 0.076 | 0.100 | 0.080 | 0.024† | 0.002† | 0.011† | 0,000 | 0,009† | 0,310 | 0,291 | 0,289 | 0,411 | 0,330 | 0,368 | 0,460 | 0,449 | 0,507 | 0,489 | 0,396 | 0,436 | 0,393 | 0,447 | 0,403 | 0,507 | 0,525 | 0,540 | 0,620 | 0,432 | 0,485 | 0,491 |
| CERs | 0.727 | 0.519 | 0.783 | 0.556 | 0.067 | 0.097 | 0.096 | 0.015† | 0.011† | 0.012† | 0.006† | 0,000 | 0,340 | 0,326 | 0,322 | 0,441 | 0,363 | 0,410 | 0,500 | 0,488 | 0,553 | 0,511 | 0,427 | 0,481 | 0,416 | 0,467 | 0,427 | 0,535 | 0,557 | 0,565 | 0,657 | 0,463 | 0,516 | 0,526 |
| TULw | 0.410 | 0.533 | 0.789 | 0.927 | 0.783 | 0.839 | 0.893 | 0.751 | 0.677 | 0.755 | 0.729 | 0.769 | 0,000 | 0,041 | 0,016 | 0,305 | 0,214 | 0,151 | 0,232 | 0,231 | 0,501 | 0,477 | 0,279 | 0,347 | 0,269 | 0,346 | 0,315 | 0,403 | 0,431 | 0,470 | 0,540 | 0,343 | 0,390 | 0,414 |
| TULal | 0.483 | 0.641 | 0.775 | 0.832 | 0.713 | 0.765 | 0.814 | 0.713 | 0.657 | 0.733 | 0.682 | 0.728 | 0.065 | 0,000 | 0,016 | 0,310 | 0,204 | 0,124 | 0,201 | 0,195 | 0,478 | 0,451 | 0,302 | 0,359 | 0,284 | 0,351 | 0,323 | 0,410 | 0,433 | 0,465 | 0,538 | 0,320 | 0,392 | 0,407 |
| TULap | 0.430 | 0.540 | 0.775 | 0.887 | 0.737 | 0.804 | 0.804 | 0.704 | 0.647 | 0.732 | 0.685 | 0.736 | 0.038 | 0.030 | 0,000 | 0,280 | 0,196 | 0,140 | 0,234 | 0,234 | 0,478 | 0,457 | 0,295 | 0,350 | 0,276 | 0,341 | 0,324 | 0,407 | 0,421 | 0,462 | 0,530 | 0,326 | 0,388 | 0,404 |
| BERw | 0.659 | 0.589 | 0.850 | 0.828 | 0.602 | 0.606 | 0.804 | 0.539 | 0.495 | 0.517 | 0.540 | 0.542 | 0.370 | 0.404 | 0.318 | 0,000 | 0,224 | 0,343 | 0,462 | 0,498 | 0,663 | 0,666 | 0,489 | 0,579 | 0,461 | 0,505 | 0,482 | 0,574 | 0,596 | 0,620 | 0,693 | 0,518 | 0,558 | 0,585 |
| TOLs | 0.605 | 0.690 | 0.826 | 0.769 | 0.671 | 0.680 | 0.748 | 0.681 | 0.622 | 0.645 | 0.618 | 0.632 | 0.404 | 0.388 | 0.374 | 0.167 | 0,000 | 0,263 | 0,314 | 0,356 | 0,565 | 0,594 | 0,396 | 0,475 | 0,364 | 0,434 | 0,413 | 0,482 | 0,527 | 0,561 | 0,643 | 0,400 | 0,482 | 0,499 |
| CERw | 0.616 | 0.710 | 0.756 | 0.788 | 0.749 | 0.748 | 0.779 | 0.721 | 0.675 | 0.708 | 0.653 | 0.704 | 0.225 | 0.163 | 0.155 | 0.270 | 0.297 | 0,000 | 0,182 | 0,213 | 0,621 | 0,623 | 0,381 | 0,465 | 0,360 | 0,434 | 0,388 | 0,489 | 0,537 | 0,569 | 0,662 | 0,401 | 0,468 | 0,497 |
| CERap | 0.632 | 0.762 | 0.806 | 0.782 | 0.775 | 0.771 | 0.771 | 0.746 | 0.710 | 0.730 | 0.683 | 0.723 | 0.262 | 0.204 | 0.272 | 0.356 | 0.260 | 0.118 | 0,000 | 0,069† | 0,692 | 0,701 | 0,506 | 0,596 | 0,457 | 0,532 | 0,488 | 0,575 | 0,621 | 0,644 | 0,720 | 0,507 | 0,559 | 0,591 |
| CERam | 0.609 | 0.850 | 0.788 | 0.788 | 0.751 | 0.759 | 0.747 | 0.736 | 0.736 | 0.750 | 0.723 | 0.732 | 0.317 | 0.228 | 0.287 | 0.456 | 0.362 | 0.158 | 0.027 | 0,000 | 0,746 | 0,760 | 0,475 | 0,587 | 0,435 | 0,521 | 0,470 | 0,575 | 0,627 | 0,657 | 0,755 | 0,494 | 0,551 | 0,597 |
| LIBam | 0.584 | 0.833 | 0.958 | 0.779 | 0.645 | 0.735 | 0.546 | 0.493 | 0.523 | 0.531 | 0.508 | 0.512 | 0.645 | 0.582 | 0.592 | 0.644 | 0.500 | 0.593 | 0.591 | 0.602 | 0,000 | 0,877 | 0,654 | 0,799 | 0,554 | 0,632 | 0,591 | 0,691 | 0,719 | 0,757 | 0,840 | 0,633 | 0,674 | 0,731 |
| POTag | 0.451 | 0.816 | 0.949 | 0.593 | 0.410 | 0.468 | 0.499 | 0.443 | 0.474 | 0.526 | 0.475 | 0.403 | 0.601 | 0.508 | 0.557 | 0.650 | 0.597 | 0.587 | 0.592 | 0.597 | 0.499 | 0,000 | 0,654 | 0,838 | 0,553 | 0,643 | 0,583 | 0,705 | 0,713 | 0,745 | 0,854 | 0,637 | 0,673 | 0,745 |
| HUJw | 0.317 | 0.657 | 0.494 | 0.945 | 0.690 | 0.622 | 0.865 | 0.741 | 0.738 | 0.752 | 0.715 | 0.749 | 0.539 | 0.653 | 0.651 | 0.691 | 0.657 | 0.611 | 0.673 | 0.660 | 0.721 | 0.757 | 0,000 | 0,197 | 0,100 | 0,248 | 0,181 | 0,319 | 0,399 | 0,433 | 0,533 | 0,280 | 0,354 | 0,371 |
| HUJag | 0.453 | 0.570 | 0.461 | 0.774 | 0.286 | 0.422 | 0.433 | 0.366 | 0.336 | 0.339 | 0.265 | 0.345 | 0.626 | 0.645 | 0.619 | 0.556 | 0.588 | 0.630 | 0.658 | 0.626 | 0.604 | 0.535 | 0.235 | 0,000 | 0,185 | 0,385 | 0,317 | 0,309 | 0,415 | 0,442 | 0,639 | 0,404 | 0,332 | 0,431 |
| PELw | 0.378 | 0.756 | 0.420 | 0.902 | 0.770 | 0.737 | 0.938 | 0.881 | 0.855 | 0.896 | 0.871 | 0.872 | 0.479 | 0.591 | 0.585 | 0.773 | 0.650 | 0.660 | 0.684 | 0.692 | 0.676 | 0.712 | 0.117 | 0.276 | 0,000 | 0,123 | 0,154 | 0,227 | 0,301 | 0,360 | 0,435 | 0,297 | 0,272 | 0,301 |
| ELOap | 0.559 | 0.854 | 0.572 | 1.000 | 0.725 | 0.715 | 0.967 | 0.785 | 0.799 | 0.788 | 0.789 | 0.773 | 0.675 | 0.677 | 0.653 | 0.653 | 0.665 | 0.635 | 0.741 | 0.731 | 0.714 | 0.779 | 0.247 | 0.333 | 0.126 | 0,000 | 0,237 | 0,402 | 0,453 | 0,483 | 0,567 | 0,377 | 0,410 | 0,436 |
| SANap | 0.510 | 0.791 | 0.630 | 0.978 | 0.730 | 0.806 | 0.852 | 0.741 | 0.692 | 0.773 | 0.722 | 0.756 | 0.647 | 0.701 | 0.731 | 0.754 | 0.736 | 0.611 | 0.666 | 0.666 | 0.706 | 0.659 | 0.241 | 0.389 | 0.239 | 0.279 | 0,000 | 0,366 | 0,412 | 0,419 | 0,506 | 0,314 | 0,379 | 0,383 |
| SJAw | 0.560 | 0.795 | 0.463 | 0.907 | 0.719 | 0.748 | 0.954 | 0.889 | 0.857 | 0.853 | 0.880 | 0.877 | 0.610 | 0.697 | 0.704 | 0.732 | 0.583 | 0.691 | 0.630 | 0.648 | 0.682 | 0.764 | 0.270 | 0.402 | 0.205 | 0.427 | 0.367 | 0,000 | 0,257 | 0,300 | 0,400 | 0,392 | 0,244 | 0,321 |
| EAGam | 0.603 | 0.851 | 0.539 | 0.997 | 0.871 | 0.832 | 0.924 | 0.845 | 0.841 | 0.872 | 0.850 | 0.865 | 0.625 | 0.654 | 0.617 | 0.761 | 0.713 | 0.727 | 0.764 | 0.769 | 0.601 | 0.630 | 0.399 | 0.454 | 0.304 | 0.490 | 0.451 | 0.140 | 0,000 | 0,408 | 0,492 | 0,440 | 0,275 | 0,309 |
| BATs | 0.617 | 0.857 | 0.552 | 0.894 | 0.891 | 0.812 | 0.926 | 0.899 | 0.894 | 0.909 | 0.855 | 0.822 | 0.771 | 0.768 | 0.773 | 0.805 | 0.755 | 0.743 | 0.768 | 0.771 | 0.775 | 0.651 | 0.363 | 0.406 | 0.335 | 0.488 | 0.417 | 0.130 | 0.221 | 0,000 | 0,488 | 0,434 | 0,372 | 0,428 |
| MAUw | 0.695 | 0.831 | 0.564 | 1.000 | 0.977 | 0.861 | 0.986 | 0.967 | 0.967 | 0.965 | 0.954 | 0.959 | 0.766 | 0.776 | 0.771 | 0.816 | 0.761 | 0.764 | 0.774 | 0.779 | 0.770 | 0.779 | 0.372 | 0.498 | 0.378 | 0.508 | 0.422 | 0.140 | 0.209 | 0.149 | 0,000 | 0,520 | 0,445 | 0,508 |
| TEMam | 0.643 | 0.942 | 0.594 | 0.780 | 0.714 | 0.702 | 0.771 | 0.764 | 0.769 | 0.769 | 0.745 | 0.754 | 0.628 | 0.534 | 0.577 | 0.670 | 0.538 | 0.489 | 0.558 | 0.510 | 0.584 | 0.592 | 0.296 | 0.386 | 0.426 | 0.391 | 0.372 | 0.362 | 0.437 | 0.360 | 0.303 | 0,000 | 0,416 | 0,409 |
| MOCw | 0.658 | 0.845 | 0.406 | 0.886 | 0.977 | 0.837 | 0.978 | 0.963 | 0.909 | 0.984 | 0.924 | 0.956 | 0.717 | 0.743 | 0.753 | 0.828 | 0.766 | 0.687 | 0.718 | 0.721 | 0.779 | 0.779 | 0.401 | 0.450 | 0.325 | 0.485 | 0.419 | 0.139 | 0.203 | 0.247 | 0.228 | 0.481 | 0,000 | 0,235 |
| MAZal | 0.683 | 0.758 | 0.467 | 0.898 | 0.823 | 0.726 | 0.846 | 0.820 | 0.826 | 0.832 | 0.808 | 0.823 | 0.710 | 0.693 | 0.704 | 0.739 | 0.688 | 0.623 | 0.641 | 0.638 | 0.659 | 0.685 | 0.313 | 0.364 | 0.317 | 0.450 | 0.374 | 0.157 | 0.128 | 0.204 | 0.159 | 0.336 | 0.136 | 0,000 |

**Table S1.** Values of the fixation index *F_ST_* (above the diagonal) and and *D*_est_ distance values (below the diagonal) between pairs of Mexican populations of *Capsicum annuum* var. *glabriusculum* from Mexico.
