# Supplementary material for: Impact of Human Management on the Genetic Variation of Wild Pepper, Capsicum annuum var. glabriusculum
Source: PLoS One. 2011 Dec 6;6(12):e28715. doi: 10.1371/journal.pone.0028715 (PMC3232243; doi:10.1371/journal.pone.0028715)

Fig.S2

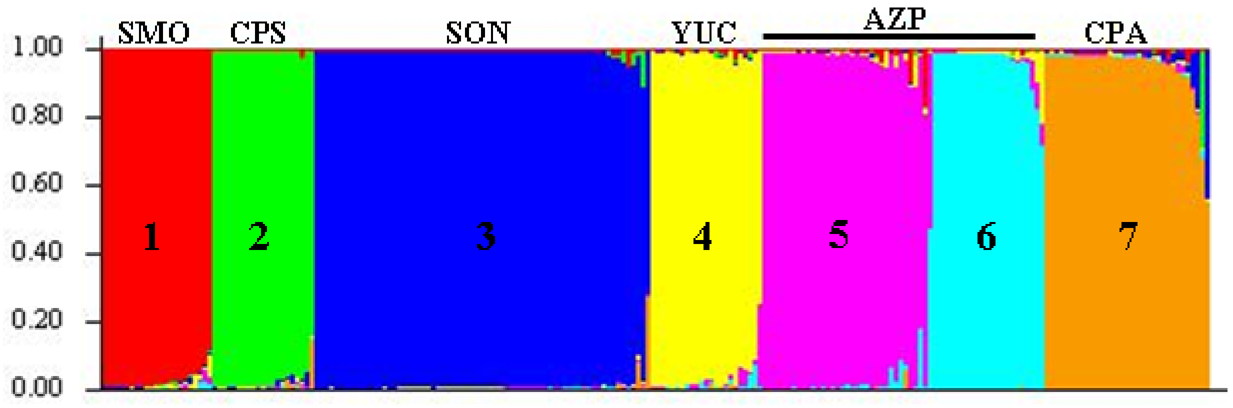

Supplement: Figure S2 — Genetic structure of wild C. annuum var. glabriusculum populations. Two hundred and twenty eight genotyped individuals clustered into 7 groups. Each individual is represented by a vertical bar, which is divided into 7 coloured fractions representing the estimated portion of its genome that assigns the individual to each of 7 clusters. Black lines separate different clusters. Biogeographical provinces are indicated at the top of the chart. (PDF) [file pone.0028715.s002.pdf]
